# Supplementary material for: Prolactin Rescues Immature B Cells from Apoptosis-Induced BCR-Aggregation through STAT3, Bcl2a1a, Bcl2l2, and Birc5 in Lupus-Prone MRL/lpr Mice
Source: Cells. 2021 Feb 4;10(2):316. doi: 10.3390/cells10020316 (PMC7913714; doi:10.3390/cells10020316)
Supplement: Supplementary file 1 [file cells-10-00316-s001.pdf]

Prolactin Rescues Immature B cells from Apoptosis-Induced BCR-Aggregation through STAT3, Bcl2a1a, Bcl2l2, and Birc5 in Lupus-Prone MRL/lpr Mice

Rocio Flores-Fernández, Angélica Aponte-López, Mayra C. Suárez Arriaga, Patricia Gorocica-Rosete, Alberto Pizaña-Venegas, Luis Chávez-Sanchéz, Francico Blanco-Favela, Ezequiel M. Fuentes-Pananá, Adriana K Chávez-Rueda.

Table S1. Gene expression among the set of housekeeping genes included in the array.

GENE	B6-Medium	B6-PRL	LPR-Medium	LPR-PRL	LPR-PRLinh	LPR-Stattic	Normfinder stability value
Actb	20.9	21.58	22.57	22.39	21.73	22.37	1.078
B2M	24.28	25.38	22.16	21.7	21.24	23.3	1.286
GAPDH	28.43	29.82	26.87	25.14	26.05	27.8	**0.753**
GUSB	23.78	24.73	29.93	29.41	29.73	30.47	4.653
Hsp90ab1	32.16	34.1	24.42	24.28	23.87	26.51	4.17
Arithmetic Mean (AM)	25.91	27.12	25.19	24.58	24.52	26.09	
Geometric Mean (GM)	25.62	26.78	25.03	24.44	24.33	25.92	

NormFinder V20 software was used to identify the optimal normalization genes among the set of housekeeping genes included in the array. Based on this analysis, the GAPDH was the housekeeping gene used to normalize the genes expression.

Table S2. Ct values of genes Bcl2l1, Bcl2l2, Birc5, and Bcl1a2a after immunoprecipitation with anti-pSTAT3 and IgG isotype control of immature B cells from 9-week-old C57BL/6 and MRL/lpr mice and WEHI-231 cells after 1 h stimulation with PRL.

	WEHI-231		C57BL/6		MRL/lpr	
	Ct1	Ct2	Ct1	Ct2	Ct1	Ct2
			Bcl2l1			
anti-pSTAT3	28.8	29.52	29.85	29.82	NA	NA
IgG	30.73	31.25	31.35	31.12	31.35	31.12
Non-template	NA	NA	NA	NA	NA	NA
			Bcl2l2			
anti-pSTAT3	29.18	29.47	NA	NA	29.30	29.22
IgG	30.73	31.25	31.35	31.12	31.35	31.12
Non-template	NA	NA	NA	NA	NA	NA
			Birc5			
anti-pSTAT3	NA	NA	29.61	29.57	28.65	28.55
IgG	30.73	31.25	31.35	31.12	31.35	31.12
Non-template	NA	NA	NA	NA	NA	NA
			Bcl1a2a			
anti-pSTAT3	NA	NA	NA	NA	28.7	28.58
IgG	30.73	31.25	31.35	31.12	31.35	31.12
Non-template	NA	NA	NA	NA	NA	NA

The PCR non-template control to each condition is also shown.

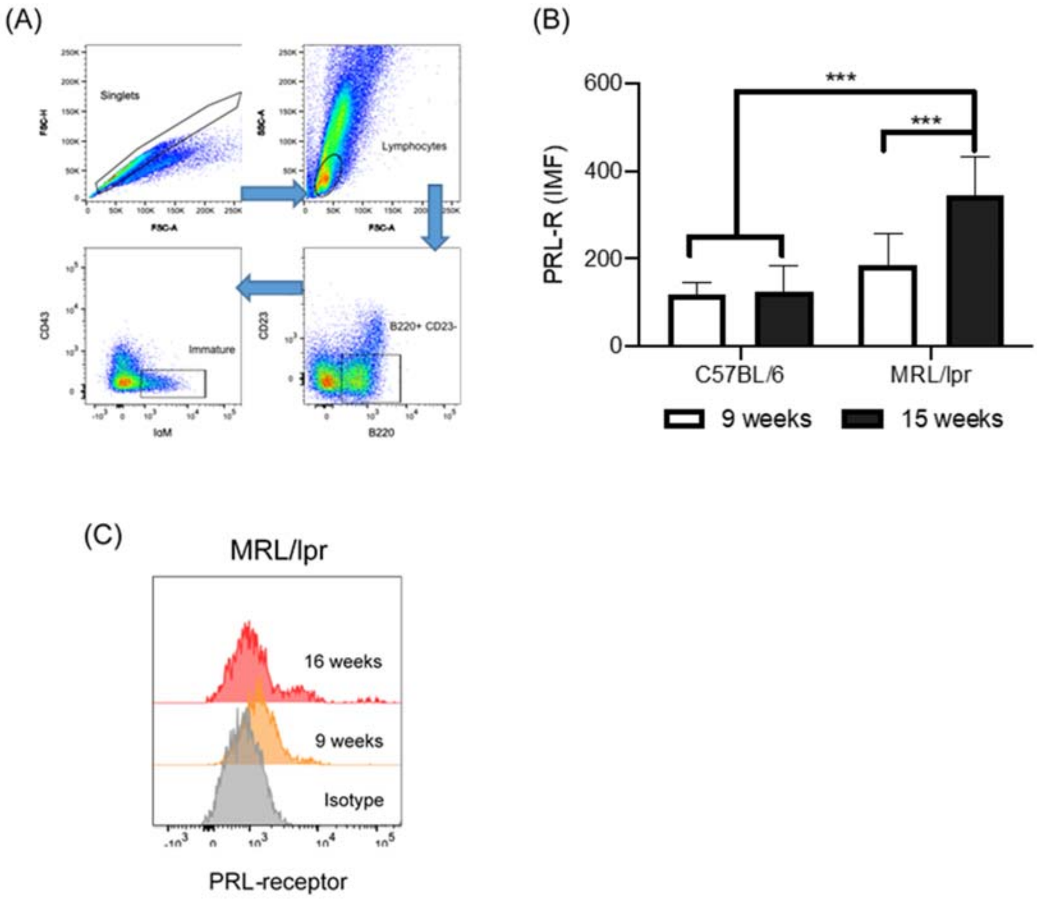

Figure S1. PRL receptor expression in immature B cells. Bone marrow cells from 9 and 15-week-old C57BL/6 and MRL/lpr mice were stained with anti-B220, anti-CD23, anti-CD43, anti-IgM, and anti PRL receptor (clone T6, Novus Biologicals, USA), to subsequently determine the median fluorescence intensity (MFI) of PRL receptor in immature B cells (B220$^+$CD23$^-$CD43$^-$IgM$^+$). (**A**) Demonstration of the gating strategy for the flow cytometric analysis of PRL receptor in immature B cell. Doublets were excluded by gating on FSC-H x FSC-A, lymphocytes were identified by their scatter properties (FSC-A x SSC-A plot). The gate of B220 B cells was selected, we excluded all mature recirculating B cells with the CD23 negative gate. The surface CD43$^-$IgM$^+$ population represents immature B-cells. (**B**) Graphic and (**C**) Histogram from one experiment representative of PRL receptor in MRL/lpr immature B cells. Three different experiments were performed, each experiment in triplicate. Pooled data are presented as mean ± SD. *** $p < 0.005$ using one-way analysis of variance (ANOVA).

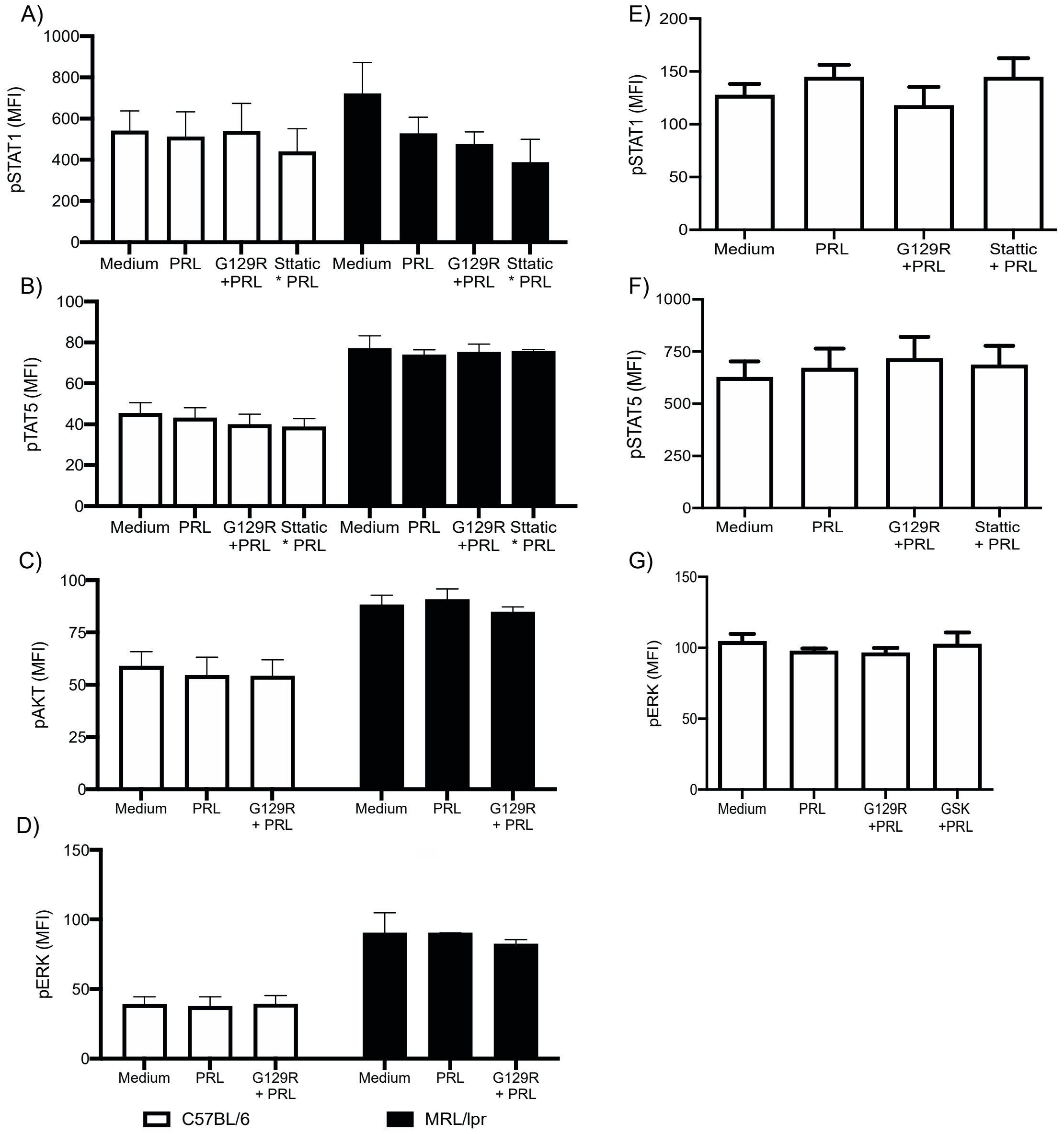

Figure S2. Analysis of the signaling pathways activated by PRL in immature B cells and WEHI-231 cells. Bone marrow B220^{+}CD23^{-} cells from 9-week-old C57BL/6 and MRL/lpr mice were pre-incubated with an inhibitor of the PRL-receptor (G129R) or Sttatic. The cells were then incubated with PRL and stained with anti-CD43, and anti-IgM, to subsequently determine the phosphorylation of **(A)** pSTAT1, **(B)** pSTAT5, **(C)** pAKT and **(D)** pERK in immature B cells (CD43^{-}IgM^{+}). Three different experiments were performed, and each experiment was conducted in triplicate. Pooled data are presented as mean ± standard deviation (SD). WEHI-231 cells were pre-incubated with the inhibitors G129R, and then incubated with PRL to determine the phosphorylation of **(E)** pSTAT1, **(F)** pSTAT5, and **(G)** pERK. Four different experiments were performed, each experiment in triplicate. Pooled data are presented as mean ± SD.
